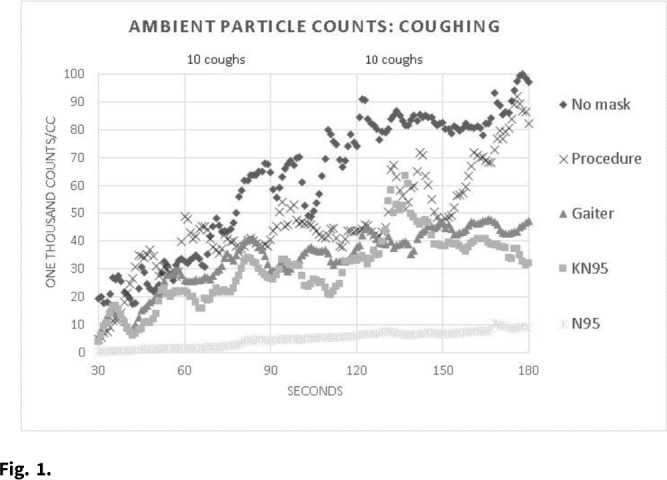# Fitted containment efficiency of face masks for reducing emission of aerosols in the indoor environment

**DOI:** 10.1017/ash.2022.132

**Published:** 2022-05-16

**Authors:** William Bennett, Steven Prince, Kirby Zeman, James Samet

## Abstract

**Background:** Face masks are a major tool for reducing the spread of COVID-19 has been the use of face masks because (1) they protect the wearer from aerosol laden virus in the environment and (2) they reduce aerosol emissions to the environment from infected individuals. Methods that quantify the fitted mask filtration efficiency for protection of the wearer are well established (eg, Sickbert-Bennett et al, *JAMA Intern Med* 2020;180:1607). In contrast, current methods for assessing face-mask containment efficiency are generally semiquantitative and rely on measurement of a very low concentration of aerosols emitted from a healthy or infected human, or the use of mannequins in which a high concentration of surrogate aerosols can be introduced inside the mask. **Methods:** Expanding on our standard methods used for fitted face-mask filtration efficiency, we designed a small-volume, low-ventilation chamber to accommodate a seated study participant. The study participant wore a ported face mask to allow introduction of a stream of 0.05 μm NaCl particles at a constant concentration (TSI 8026 particle generator) into the mask space. The ambient chamber concentration was continuously measured by a TSI 3775 condensation particle counter sampling 2 feet (~2 m) in front of the participant’s head over a series of three 3-minute periods: (1) resting, (2) reading out loud, and (3) repeated forceful coughing (2 × 10 coughs) (~450 L/min peak flows). Figure 1 shows a raw data sample for the coughing procedure. Containment efficiency (%) for each mask and procedure were determined as 100 × (1 – the average of all 1 − second ambient concentration values between 30 and 180 seconds divided by the same for the “no mask” condition). **Results:** Table 1 shows the average % containment efficiency for 2 study days with each mask or procedure in an adult male. The 2-ear-loop masks (KN95 and procedure) tested during coughing had the greatest reduction in % containment efficiency compared to that during resting breathing, likely owing to a decreased mask fit with transient pressure increase inside the mask associated with the coughs. The N95 was least affected by the introduction of reading and/or coughing, maintaining near 95% containment efficiency throughout. **Conclusions:** Our preliminary data on fitted containment efficiency of masks under different conditions suggest that the fitted containment efficiency closely mimics their performance for personal protection. This information that may aid in providing optimum source control in indoor environments.

**Funding:** None

**Disclosures:** None

**Table:**
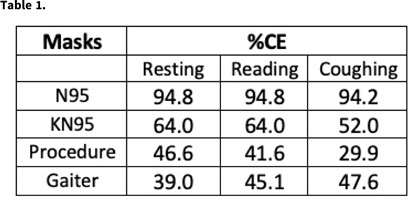

**Figure:**